# The effects of economic stress and urbanization on driving behaviours of Boda-boda drivers and accidents in Gulu, Northern Uganda: a qualitative view of drivers

**DOI:** 10.11604/pamj.2020.36.47.21382

**Published:** 2020-06-01

**Authors:** David Lagoro Kitara, Omar Karlsson

**Affiliations:** 1 Harvard University, Harvard T.H. Chan School of Public health, Department of Global Health and Population,Boston, Massachusetts, United States of America; 2 Gulu University, Faculty of Medicine, Department of Surgery, Gulu, Uganda; 3 Lund University, Department of Economic History and Demography, Lund, Sweden

**Keywords:** Gulu Municipality, Boda-boda drivers, accidents, economic stress, Uganda

## Abstract

**Introduction:**

Understanding motorcyclists’ on-the-road behaviour is critical for developing and evaluating interventions specifically targeting them. Risky on-the-road behaviours have historically been subdivided into errors and violations of the rules of road use. Police records could be used to record cyclists’ behaviours, however these documents do not necessarily capture all errors and violations. None use of protective wears have been documented in many studies conducted on Boda-boda drivers in Uganda. The relationship between drivers’ economic stress and road safety have been studied for many years but the effects of global and economic stress, its joint effects on behaviours of drivers in relations to accidents have received very little attention. This study aimed at assessing the relationship between injuries and the Boda-boda drivers’ behaviours in Northern Uganda.

**Methods:**

Two hundred Boda-boda drivers from Gulu Municipality had face-to-face interviews to assess views and underlying factors on the issue of high prevalence of motorcycle road traffic accidents and injuries. Descriptive statistics were used to describe factors on road accidents including personal information, knowledge, skills and attitudes towards road use and safety. The study was approved by a local Institutional Review Board (IRB).

**Results:**

Collectively, the results showed that economic stress is an important factor that affects drivers’ behaviour and increases their risks to motorcycle accidents.

**Conclusion:**

These authors suggest that more studies should be conducted to determine the relationship between economic stress, anger and dangerous behaviours among Boda-boda drivers and accidents in Gulu Municipality using a Motorcycle Rider Behaviour Questionnaire (MRBQ).

## Introduction

Psychometric research within road traffic safety has for a long time produced various questionnaires and scales for measurement of risky drivers’ behaviours and similar constructs have been made with the intention of predicting individual differences in safety [1]. Understanding motorcyclists’ on-road behaviour is critical for developing and evaluating interventions specifically targeting them [1]. Risky on the road behaviours have historically been subdivided into errors and violations of the rules on road use [2]. Police records are documents that could be relied on, to record behaviours of cyclists [2]. Unfortunately, in most low-to-middle income countries, these documents do not necessarily capture all errors and violations of drivers due to variability in enforcement and because in most jurisdictions and countries the use of safety equipment is not mandated except for helmets [2]. None use of protective wears such as helmets, goggles and gloves have been documented in many studies conducted (for example; self-reporting, face-to-face interviews, direct observations, official records of Ugandan police and hospitals records where the studies were conducted) on Boda-boda drivers in Uganda and targeted interventions to improve their use have not measured to the expectations of the framers of the policies [3-6]. Stress is a modern phenomenon that affects almost everyone in their daily lives [1]. It is most commonly conceptualized as the body’s response to any demand that exceeds individual’s adaptive capacity [7, 8]. In addition, stress has been shown in many studies that; it affects attention [9], anxiety [10, 11] and working memory [12]. It has further been observed that stress affects cognitive neuroscience and that stress hormones affect pre-frontal brain regions and dopaminergic pathways [13, 14]. As a common daily activity for many people, driving behaviour is affected by various stressors [15, 16]. Surprisingly, very few and limited studies have explored the relationships between driving-related stressors, road safety and road accidents [16]. This is because it has been observed that driving-related stressors arise mainly from personal and environmental factors that make drivers feel stressed when they are driving [16]. Therefore, drivers’ stress is an important factor in research in determining the relationship between driving-related stressors, accidents and driving behaviours [17].

It has been noted in some studies that, drivers’ stress mostly occur when an individual perceives that his driving ability is insufficient, to manage the demands, and to handle the dangers of driving, and thus induce a dislike for driving and hazard monitoring [15, 18]. In addition, dislike of driving encompasses a driver’s feeling of anxiety, tension, frustration, joylessness and lack of confidence especially in complicated driving situations [19]. This author further argue that dislike for driving is associated with diminished control skills in simulated driving tasks [19]. Other studies have revealed that high levels of drivers’ stress are associated with self-reported mistakes and violations during driving [20,21], and that, the other factor related to it is, the driving environment which generates stressors related to the time pressure, congestion and road conditions [22-24]. Furthermore, stressors related to driving and driving environment could be exogenous to the driving situations and these include; life events, personal situations, daily frustrations which may influence driving behaviours and occurrence of traffic violations and accidents [17]. In this paper, we assessed factors that are associated with frequent road traffic accidents with Boda-boda drivers in Gulu Municipality; a rapidly growing city but with a large section of its Boda-boda drivers economically frustrated by pressures of rapid urbanization and poor daily incomes. We observed that whereas the Boda-boda drivers have adequate knowledge on road safety measures and have been trained on many occasions, with correct attitudes on road use, many are involved in road accidents mainly in part because of poor daily incomes from Boda-boda driving in an environment of many economic challenges.

## Methods

**Study design:** a cross-sectional study was conducted in four divisions of Gulu Municipality, Uganda in 2015.

**Study settings:** the study was conducted at the Boda-boda road-side stages in (Laroo, Bardege, Pece and Layibi divisions) of Gulu Municipality, Northern Uganda.

**Study participants:** Boda-boda drivers who were registered and were already operating at Gulu Municipality road-side stages three months prior to this study. Written informed consent was a requirement for enrolment.

**Study instrument:** we used questionnaires to conduct face-to-face interviews with two hundred out of six hundred registered Boda-boda drivers in Gulu Municipality. All 200 Boda-boda drivers completed the interviews (100% completion rate). The questionnaire was constructed in two major parts: Structured and unstructured questions. The first part contained; socio-demographic characteristics of the drivers and questions on road traffic accidents, knowledge on road safety measures, attitudes and skills in Boda-boda driving. The second part contained questions which were open handed and gave the drivers the opportunity to express their views, thoughts and opinions freely with minimal limitations. All observations were summarized into descriptive statistics with numbers and percentages. The questions in the questionnaire had an internal validity Cronbach’s alpha coefficient of more than 0.73 (Table 1).

**Table 1:** Internal validity of the variables

Variables	Number of items	Cronbach's alpha	Mean
Knowledge on road use	152	0.72	0.874
Getting trained first before driving Boda-boda	118	0.74	0.621
Have good attitudes towards driving	157	0.76	0.882
Use of helmets	167	0.73	0.944

The questions in the questionnaire had an internal validity Cronbach’s alpha coefficient of more than 0.73

**Ethical approval:** this study was approved by a local IRB and Uganda National Council of Science and Technology (UNCST). Each participant gave a written informed consent and their personal information was kept confidential. We ensured Privacy of Boda-boda drivers at a distance in, the quiet of their road-side stages. Records of the interviews were maintained and kept anonymous, ensuring that confidentiality of personal information was carefully and properly maintained.

**Data analysis:** data was analyzed in two parts: We summarized their qualitative answers into themes with numbers, and percentages and where required, STATA software version 14.1 was used for further statistical analysis.

## Results

In summary, the socio-demographic characteristics of Boda-boda drivers were, sex, 198(99.0%) males and 2(1.0%) females, mean age 29(SD+13.6) years, and age ranged from 16-48 years. The majority were; Catholics (58.5%), Acholi (87.5%), Boda-boda, the main occupation (89.5%), certificate, the highest level of education (46.0%), mainly from Bardege division (28.5%), Single (not married) (50.0%), owned their motorcycles (50.5%); and had work experience of 5-9 years (52%). A total of 180(90%) drivers reported the years of work experience ranging from 1 to 14 years. A total of 104(53.6%) drivers reported to have been involved in road traffic accidents in the last one year; majority (77.8%) earned less than $5 a day; (54.7%) had a child and (79.5%) of those had more than two children; (71.5%) had no house and (70%) could not afford to pay rent and lived with friends or relatives; of those renting, most (98.3%) could afford a monthly house rent less than $12.5; (68.3%) of the houses had no running water and (67.8%) no electricity. Where there was electricity (32.2%), the majority (53.3%) could only afford less than UGX20,000/= [equivalent of $5.3] per month. Similarly, for running water (31.7%), the majority (81.3%) could only afford less than UGX20,000/= [equivalent of $5.3] per month (Table 2). The majority of drivers begin their work between 6:00am-7:00am EST in the morning and end between 8:00pm-9:00pm EST in the evening (this is approximately 14 hours of driving) (Table 2). Only (75.4%) had driving permit, (70.0%) were trained and tested on road safety measures, (80%) knew the road safety rules, (87.9%) had helmets but only (78.9%) wore them all the times while on duty. (80.5%) had other protective wears, (62.6%) agreed that they were the main cause of accidents in Gulu Municipality (Table 3), (62.1%) had regular training on road safety measures, (85%) agreed they should first undergo mandatory formal training in driving before being allowed to drive Boda-boda.

**Table 2:** Descriptive statistics for Boda-boda drivers in Gulu Municipality

Variables	Frequence (n)	%	Response Rate %
**Marital status (Single)**	100	50.0	
Others (Married, cohabiting, divorced, widowed or separated)	100	50.0	100.0
Yes, I do have electricity in my house	64	32.2	
No, I do not have electricity in my house	135	67.8	99.5
**How much do you pay for electricity per month?**			
Less than 20,000/=	34	53.1	
More than 20,000/=	30	46.9	100.0
Yes, I do have running water in my house	63	31.7	
No, I don't have running water in my house	136	68.3	100.0
**How much do you pay for water per month?**			
Less than 20,000/=	39	81.3	
More than 20,000/=	9	18.8	100.0
Yes, I do have a driver's permit	150	75.4	99.5
**What do you use since you do not have a driver’s permit?**			
I drive without	43	87.8	
I use a friend's permit	6	12.2	100.0
Yes, I had formal training on driving	133	70.0	
No, I didn't have any formal training	57	30.0	95.0
**Where did you learn driving Boda-boda from?**			
On the streets	10	7.5	
From a driving school	92	69.2	
Others (Guest house, no training, at the Boda-boda stage)	31	23.3	100.0
Yes, I do own a helmet	157	87.2	95.0
Yes, I do have other protective wears	160	80.0	100.0
**Why don't you have protective wears?**			
It is expensive to buy	15	88.2	
I lack money and cannot afford	2	11.8	42.5
**At what time do you begin your work daily?**			
6:00am-7:00am	160	82.9	
Others (5:00am, 8:00am, 9:00am)	33	17.1	100.0
**At what time do you end your work daily?**			
8:00pm-9:00pm	94	49.5	
Others (10:00pm, 11:00pm, 12:00am, 1:00am, 2:00am)	96	50.5	100.0

**Table 3:** Qualitative responses on attitudes by Boda-boda drivers in Gulu Municipality

Variables	Freq (n)	%	Response rate %
I disagree that Boda-boda drivers are the main cause of accidents in Gulu Municipality	65	37.4	
I agree that Boda-boda drivers are the main cause of accidents in Gulu Municipality	109	62.6	87.0
**The reasons why I agree?**			
They drive Boda-boda when they are drunk	27	22.1	
They lack formal training in driving Boda-boda	38	31.1	
They are always over-speeding their Boda-boda	21	17.2	
They do not concentrate on their work	17	13.9	
Others (overloading, road congestions, many pedestrians, no road signs)	19	15.6	70.1
**Reasons why I disagree?**			
Pedestrians do not know how to cross roads in Gulu town	18	41.9	
There are many other reckless road users in Gulu town	6	14.0	
Others (Poor road conditions, pedestrians do not know how to cross roads in the town)	19	44.2	66.2
**When you caused an accident, what was the circumstance?**			
I was over-speeding with my Boda-boda	34	32.7	
I was knocked by another Boda-boda	22	21.2	
The road surface was very bad	19	18.3	
Others (knocked by a car, I was tired and sleepy and was overtaking a vehicle)	29	27.9	100.0
**What did you do immediately after the accident happened?**			
I carried the victim to the Hospital	75	72.1	
I rode away	16	15.4	
Others (Removed my protective wears, arrested the other driver, couldn’t do anything since I was injured)	11	10.6	100.0
**Why did you respond in that way after the accident?**			
The victim had injuries which required treatment	63	70.0	
We need to save lives of victims of accidents	13	14.4	
Others (I was not injured, I feared the police would arrest me)	14	15.6	86.5
**Why do you help people who get involved in accidents?**			
To save life and rescue the situation	115	67.6	
Accident can lead to bleeding and death	26	15.3	
It helps in filing a case in police	10	5.9	
Others (To avoid loss of life, so that in the future I can also be helped)	19	11.2	85.0
**How long did it take you to report to police in this case of an accident?**			
2 hours	55	31.8	
1 hour	40	23.1	
I do not report	2	1.2	
Others (Immediately, sometimes depends on circumstance, 4 hours, 3 hours)	76	43.9	86.5
**Why do you report quickly to police when someone gets an accident?**			
It promotes understanding between the people involved in the accident	5	3.3	
It helps the police to investigate the case thoroughly	11	7.3	
It helps in the treatment of the injured person	111	73.5	
Others (It saves lives, it helps prevents more accidents, it supports the police)	24	15.9	87.3

However, only (38.9%) believed that the police arrests those drivers without driving permit. In addition, (72%) agreed that drivers who cause accidents should be arrested by the police, and (74.6%) agreed that training of drivers would significantly help in reducing the rampant road traffic accidents in the Municipality due to poor motorcycle driving behaviours (Table 4). Most drivers (87.4%) believed that they had adequate knowledge on road safety regulations, (85.7%) would not accept a driver who had previously caused an accident to carry their relatives, (81.4%) would not accept to be carried by such a driver, and (84.1%) would not recommend that driver to carry their most trusted clients. However, the majority (93.5%) of the drivers recommended Boda-boda motorcycles as the best means of transport for Gulu Municipality (Table 4).

**Table 4:** Socio-demographics, knowledge, attitudes and behaviours of Boda-boda drivers

Variables	Mean	95% CI	No(n)	Yes(n)
Do you have a child?	0.547	[0.476, 0.619]	86	104
Do you have your own house?	0.279	[0.215, 0.343]	137	53
Do you have electricity in your house?	0.305	[0.238, 0.373]	133	56
Have you had any formal training on Boda-boda driving?	0.700	[0.634, 0.766]	57	133
Have you been trained on road safety procedures?	0.768	[0.708, 0.829]	44	146
Do you have your own helmet?	0.879	[0.832, 0.926]	23	167
Do you use your helmet all the times while driving Boda-boda?	0.789	[0.731, 0.848]	40	150
Do you have other protective wears for driving Boda-boda?	0.805	[0.748, 0.862]	37	153
Do you think Boda-boda drivers are the main cause of road accidents in Gulu Municipality?	0.365	[0.291, 0.439]	106	61
Do you know the rules that govern road use?	0.800	[0.743, 0.857]	38	152
Have you read the rules that govern road use?	0.800	[0.743, 0.857]	38	152
Are road safety rules available to the Boda-boda drivers?	0.327	[0.256, 0.399]	115	56
Are there programs for training Boda-boda drivers by government?	0.621	[0.551, 0.691]	72	118
Do you agree that it is good to be trained first before you begin driving Boda-boda?	0.621	[0.551, 0.691]	72	118
Does the police arrest Boda-boda drivers who do not have driver’s permit?	0.389	[0.320, 0.459]	116	74
Is it good for a Boda-boda driver that frequently causes accidents to continue driving?	0.746	[0.683, 0.809]	48	141
Do you agree that training every driver on road safety rules will reduce the number of accidents on our roads?	0.746	[0.683, 0.809]	48	141
Do you have knowledge on road safety regulations?	0.874	[0.826, 0.921]	24	166
Do you agree that Boda-boda drivers should always wear a helmet while driving?	0.944	[0.909, 0.978]	10	167
Would you give advice to your colleague who frequently causes accidents to be more careful?	0.882	[0.834, 0.930]	21	157
Can you accept a Boda-boda driver who previously caused an accident to carry your relatives?	0.143	[0.093, 0.193]	162	27
Can you accept a Boda-boda driver who frequently causes accidents to carry you?	0.184	[0.129, 0.240]	155	35
Would you recommend your trusted client to a Boda-boda driver who frequently causes accidents?	0.159	[0.106, 0.211]	159	30
Do you recommend Boda-boda motorcycles as the best mode of transport in Gulu Municipality?	0.935	[0.898, 0.973]	11	159
Are emergency medicines available to Boda-boda drivers?	0.016	[-0.002, 0.034]	183	3
Have you ever seen emergency drugs with Boda-boda drivers?	0.016	[-0.002, 0.034]	187	3
Do you have emergency medicines on your Boda-boda?	0.074	[0.036, 0.111]	176	14
Have you ever made recommendations for emergency medicines to your colleagues?	0.053	[0.021, 0.085]	179	10
What is your average monthly income from Boda-boda driving (‘000)?	434.1	[400.8-467.4]		
My marital status (Single)	0.500	[0.488-0.678]		
Certificate, is my highest level of education	0.346	[0.394-0.587]		
The average age of the Boda-boda drivers in Gulu Municipality (years)	29	[27.7-30.2]		
The majority of the Boda-boda drivers in Gulu Municipality has a work experience of (5-9) years	0.52	[0.313-0.677]		
The majority of the Boda-boda drivers that caused accidents were from Pece Division in Gulu Municipality	0.346	[0.26-0.444]		

**Internal validity of the instrument:** the internal validity and consistency of the questions indexed by the Cronbach’s alpha coefficient [25] were as follows for the four categories of questions (Table 1).

From Table 2, it was observed that the majority of drivers were poor and could not pay for electricity, water and protective wears. The average total work duration in 24hours was fourteen hours on average and this was considered too long for a driver to remain alert and efficient on the roads. From Table 3, there is generally a good attitude of Boda-boda drivers towards the victims of Boda-boda accidents and in many occasions, they rushed them to hospital to receive medical treatment. Some of them drive away from the scene of accidents in order to avoid being arrested or questioned by the police. Table 4 describes the socio-demographic characteristics of Boda-boda drivers in Gulu Municipality, their knowledge on road safety rules, attitudes to road use and skills in handling victims of road accidents.

## Discussion

Road traffic injuries are the eighth leading cause of death globally and the leading cause of death among young people aged 15-29 years and nearly 3,400 people die on the world's roads every day [26]. A disproportionate burden is borne by Low-and-Middle-Income Countries (LMICs) and vulnerable road users such as pedestrians, cyclists and riders of motorcycles are who the most affected [27]. Current trends suggest that by the year 2030, road traffic deaths will become the fifth leading cause of death unless urgent action is taken [28]. Interestingly, road traffic injuries are estimated to cost LMICs between 1-2% of their Gross National Products (GNP) which is estimated at over US$ 100 billion a year [29]. Furthermore, the rate of road traffic injuries in LMICs is observed to be twice as much as that in high-income countries [26].

The African region has the highest road traffic fatality rate (24.1 per 100,000 population) and it has been observed that motorcyclists are among the most vulnerable road users [26] with nearly a quarter (23%) of the world’s road traffic deaths occurring among motorcyclists [26]. In addition, it has been noted that per vehicle mile traveled, motorcycle riders have a 34-fold higher risk of death in crashes than people driving other types of motor vehicles [30]. Notably, the commonest injuries among motorcyclists are mainly the lower-extremities and head injuries [3,30]. The other accident related effects on the victims may range from minor abrasions to fractures and spinal deformations [6, 31]. According to the USA Federal Government report, it was observed that per mile traveled in 2006, there were 35 times more deaths from motorcycle accidents than from car accidents [32]. It was noted that motorcycles face higher dangers from several different road hazards than do cars and other vehicles [33]. Due to the smaller size and less stable nature of the motorcycles, potholes, dead animals, slick pavement conditions, uneven heights between lanes and other irregularities or an unexpected objects in the road, pose a serious safety threat to motorcyclists [6]. Again, because the motorcyclist is not surrounded by a metal case, it is likely to be thrown far and hard and such crashes are more deadly than those involving other vehicle types [6]. Motorcycle injuries therefore constitute a major but neglected public health problem in a rapidly motorizing LMICs and the relevant risk factors have not been adequately examined in these countries [6, 30]. Several studies conducted in several countries have established factors that have influence on motorcycle crashes including; age, ethnicity, level of education, motorcycle license and insurance status, self-reported alcohol consumption in the 12 hours preceding the crash, years of on-road riding experience, kilometers travelled on the specific motorcycle at interview, posted speed limit and weather conditions [6,34]. Other factors such as engine sizes, time of day [6], motorcycle ownership, speed and risk-taking behaviors have been documented [30]. Most factors associated with injuries among commercial motorcyclists in these countries have produced descriptive data on victims of the Boda-boda crashes with little or limited efforts to identify predisposing factors associated with the injuries [35, 36].

There is a high prevalence of road traffic accidents in Uganda due to Boda-boda drivers and particularly in Gulu, Northern Uganda. This study showed that a number of issues were associated with Boda-boda accidents and among the many were, poor daily and monthly incomes of the drivers (Table 2, Table 3). This is coupled with the high economic demands in the rapidly urbanizing Gulu town in Northern Uganda where the costs of living is relatively higher compared to other towns in Uganda [37-39]. This high costs of living is partly due to the increasing population and therefore increased demands for goods and services but also because of increasing and uncontrolled exportation of goods and services to the neighbouring country, South Sudan; a country which is facing civil unrest, there is low agricultural productivity and they mainly rely on supplies of goods and services from towns in Northern Uganda, particularly Gulu [37-39]. Interestingly, Graham, Glaister and Anderson in their study in Britain found that the incidences of child pedestrian casualties (children defined as 0-15 years of age) in the most deprived ward was 4.07 times higher than in the least deprived ward [40]. For adult pedestrian casualties, the corresponding incidence rate ratio was 2.28. For pedestrian accidents in which the pedestrian was killed or seriously injured, the incidence rate ratio between the most and least deprived ward was 4.4 for children and 2.5 for adults [40]. This finding was consistent with observations made in Gulu Municipality where, most Boda-boda accidents occurred in the most deprived divisions of Gulu Municipality. Furthermore, in a report produced by the European Transport Safety Council (ETSC) in 2007, it was observed that little is known about the socio-economic dimensions of road traffic injuries especially on the commercial motorcycle drivers [41]. However, the preponderance of evidence suggests that traffic injury is associated with social status [41]. Those who are low in social status sustain traffic injuries more often than those who are high in social status and social disparities in risk appear to apply to all groups of road users and all levels of injury severity [41]. This means that those groups of population who were disadvantaged in terms of income, education or quality of their residential areas were also disadvantaged as users of the road transport system by sustaining injury more often than the more advantaged segments of the population [41]. There is thus a significant element of social injustice with respect to traffic injury [41]. This finding is consistent with findings on Boda-boda drivers and accidents in Gulu Municipality where the majority were from one of the most deprived divisions (Pece) of the Municipality.

In addition, Boda-boda drivers work for far too long with an average of 14 hours a day, driving with very limited time for rest and no regulation on their activities (Table 2). This long hours is associated with increased fatigue, errors and sleeping while driving and with resultant road traffic accidents (Table 3). This may perhaps be a major contributing factors to the increased Boda-boda accidents in Gulu Municipality (Table 4). It was noted that whereas many drivers reported they had driving permits, some did not have and were driving without or using a friend’s permit in order to avoid being caught by the police (Table 3). Some drivers did not go through the normal and formal training processes of learning how to drive motorcycles (Table 2). In addition, many Boda-boda drivers reported that some of their colleagues had not been properly trained in driving motorcycles and that could explain in part the increasing numbers of accidents among the drivers in Gulu Municipality (Table 3, Table 4). Furthermore, there is an increasing human population, cars, motorcycles and other road users in Gulu Municipality as a result of the increasing urbanization and the anticipated attainment of Gulu, a city status in July, 2020 [37-39, 42]. This has led to congestion and competitive use of the roads in Gulu Municipality and particularly the pedestrians from rural areas who are not trained on road crossings in the town (Table 3). This could yet be another factor contributing to the increasing number of Boda-boda accidents in Gulu Municipality. Additionally, there was concern that a number of Boda-boda drivers were reckless on the roads and more so they seemed not to be attentive while driving on these busy roads (Table 3). Could fatigue, tiredness, poor daily income, long hours of driving and other social issues, contributory factor to Boda-boda behaviors and increasing road traffic accidents in Gulu Municipality? Finally, it has been reported on several occasions that many of the drivers exhibit abnormal behaviours such as: driving without permit, others borrow permit from friends, some get drunk, others use drugs, some are reckless, over-speed, over-take at wrong places, do not follow their lanes, do not follow road signs and work non-stop for over 14 hours per day (Table 2, Table 3). Perhaps many of these factors together with economic stress due to poor daily income contribute to the poor driving behaviours of Boda-boda drivers in Gulu Municipality and that may be a contributory factor in the increasing incidence of motorcycle accidents in this town.

**Funding:** this study was partly funded by the University of Oxford under the COOL project in addition to the support from individual authors of this paper.

## Conclusion

Driving safety is influenced by human factors in various ways and actions. In one part the knowledge, attitudes and behaviours of the drivers and on the other the pedestrians. These two issues together with the environmental factors markedly influence the outcome of driving especially, accidents. In addition, the everyday life stresses of drivers which are specific and require drivers’ attention are considered factors in determining the daily outcome of motorcycle driving. The effects of poor daily and monthly incomes where drivers were unable to meet their life demands, perhaps contributed to the bnormal behaviours of Boda-boda drivers in actions such as; risky over-taking, over-speeding, driving for very long hours, driving under the influence of alcohol and drugs, not respecting other road users and not following road signs. These observations highlight the need for continuous training of Boda-boda drivers and pedestrians in Gulu Municipality on rules and regulations of road safety in light of the new developments in Gulu town. In some of these trainings, the use of protective wears, road safety regulations, use of alcohol and drugs should be included in the curriculum of the training. Furthermore, Boda-boda drivers need to start other new economic activities since this motorcycle business alone cannot meet their economic demands in Gulu Municipality. It is also suggested that future studies on Boda-boda drivers in Gulu Municipality should be conducted using Motorcycle Rider Behaviour Questionnaire (MRBQ) to assess in details the relationships between drivers’ stress, anger, behaviours and accidents.

### What is known about this topic

- It’s one of the commonest cause of road traffic accidents in Uganda;
- It’s one of the cheapest, agile, affordable and convenient method of transportation;
- It is a source of livelihood for many unemployed youth.

### What this study adds

- Poor daily income contributes to the increased cases of accidents in Gulu, Northern Uganda; in addition, economic stress due to fast urbanization of Gulu town with increasing population, improved social amenities, high prizes of goods and services makes life in Gulu Municipality very competitive for the Boda-boda drivers;
- Long hours of driving unregulated contribute to fatigue, lack of concentration and accidents;
- Uninformed pedestrians from the rural areas who do not know how to cross roads in the town contribute to the increased incidences of accidents with the drivers.

## Competing interests

The authors declare no competing interests.
